# Effect of Cigarette Smoking on Risk of Hip Fracture in Men: A Meta-Analysis of 14 Prospective Cohort Studies

**DOI:** 10.1371/journal.pone.0168990

**Published:** 2016-12-30

**Authors:** Zhen-Jie Wu, Peng Zhao, Bin Liu, Zhen-Chao Yuan

**Affiliations:** 1 Department of Bone and Soft Tissue Neurosurgery, Affiliated Tumor Hospital of Guangxi Medical University, Nanning, People's Republic of China; 2 Department of Head and Neck Surgery, Affiliated Tumor Hospital of Guangxi Medical University, Nanning, People's Republic of China; Van Andel Institute, UNITED STATES

## Abstract

**Background:**

Several observational studies have suggested an association between cigarette smoking and risk of hip fracture. However, no formal systematic review or meta-analysis was performed to summarize this risk in men.

**Materials and Methods:**

A search was applied to MEDLINE, EMBASE, and web of science (up to November 1 2016). All prospective cohort studies assessing risk of hip fracture with the factor of cigarette smoking in men without language restriction were reviewed, and qualities of all included studies were assessed using the Newcastle-Ottawa Scale. Two authors independently assessed literatures and extracted information eligibility, and any disagreement was resolved by consensus. Newcastle-Ottawa quality assessment scale was used to evaluate studies’ quality in meta-analyses. We calculated the RR with 95% CIs in a random-effects model as well as the fixed-effects model using the metan command in the STATA version 12.0 (StataCorp, USA).

**Results:**

Fourteen prospective cohort studies were eligible for the present analysis. A meta-analysis of 12 prospective studies showed that the relative risk (RR) for current male smoking was 1.47 [95% confidence interval (CI) (1.28–1.66), p = 0.54; *I*^2^ = 0%]. Subgroup analyses show study characteristics (including geography region, length of follow-up, size of cohorts and study quality) did not substantially influence these positive associations. Eight studies reported the RRs for former smokers compared with never smokers and the pooled RR was 1.15 [95% CI, (0.97–1.34), (*I*^2^ = 0%, p = 0.975)].

**Conclusions:**

The present meta-analysis of 14 prospective studies suggests that, compared with never smokers, cigarette smoking increases risk of hip fracture in man, specifically in current smokers. However, further larger prospective cohorts with more power or meta-analysis of individual patient data are needed to confirm this association.

## Introduction

Hip fracture is a worldwide health issue, which is associated with a pronounced morbidity and excess mortality not only in North America but also in Asia and Europe [1]. It is suggested that the number of hip fracture in the world will increase from 1.66 million in 1990 to 6.26 million by 2050 [2].There is a demonstrated research show that approximately 19% of all hip fractures were attributed to cigarette smoking, and the relative risk (RR) for current smokers comparing with never smokers was consistently higher in male than in female [3].

Recently, a meta-analysis has suggested that there was a positive association between cigarette smoking and hip fracture in woman [4]. However there is, to our knowledge, no published meta-analysis had evaluated this association in man. Some researches demonstrated a significant positive association between cigarette smoking and risk of hip fracture in men [3, 5–7], and others did not research a significant association [8–12], and there were still some articles did not support this relationship [13].

In 2003, a meta-analysis, including cohort, case-control, and cross-sectional studies, had estimated that smoking was associated with an increased risk of hip fracture [14]. As we know, case-control studies and retrospective studies may generate bias. Therefore, we performed a meta-analysis using the data from published prospective cohort studies to evaluate the relationship between smoking and risk of hip fracture in male.

## Materials and Methods

We conducted this meta-analysis according to the PRISMA guidelines (S1 Table). We systematically searched MEDLINE, EMBASE, and web of science for prospective cohort studies which evaluating the associations between cigarette smoking and risk of hip fracture in man from their inception to November 1, 2016 without any restrictions. In brief, search terms included: ‘fracture’ OR ‘osteoporosis’ AND (smoking OR cigarette OR tobacco). In addition, a manual search of the reference lists of potential relevant and practice guidelines were performed to identify any additional studies. In case of any differences in opinions, a third reviewer was consulted.

### Study selection

The fully published studies were included only if they comprised the following criteria: (1) studies that were prospective cohorts studies designs; (2) studies reported RR or Odds ratio (OR) and their corresponding 95% confidence intervals (95% CIs) of hip fracture by different smoking categories or provided raw data to calculate these; (3) studies contained man both exposed and not exposed to smoking; and (4) data not duplicated in another article.

### Data extraction and quality assessment

Two authors (ZW and PZ) independently assessed literatures and extracted information eligibility, and any disagreement was resolved by consensus. The following data were summarized from each study: first author, study years, location, duration of follow up, size of cohort, age, number of hip fracture patients, smoking status, RR (95% CI), study quality, and adjustment for covariates. The most adjusted relative risks were selected if studies reported more than one set of adjustments. Newcastle-Ottawa quality assessment scale was used to evaluate studies’ quality in meta-analyses based on three items: patient selection, comparability of controls, and ascertainment of outcome. This quality assessment scale ranges between zero up to nine stars [15].

### Statistical analysis

We calculated the RR with 95% CIs in a random-effects model as well as the fixed-effects model using the metan command in the STATA version 12.0 (StataCorp, USA). And the Statistical heterogeneity across studies was assessed by the Q statistic test and I2 statistics test. However, we just reported the random-effect model, as it was considered to be more natural. A sensitivity analysis was also performed to eliminate each study at a time from the meta-analysis. And the Begg’s test and Egger’s test was used to assess the bias of publication while it was considered significant when p<0.05.

## Result

### Literature search

**Fig 1:**
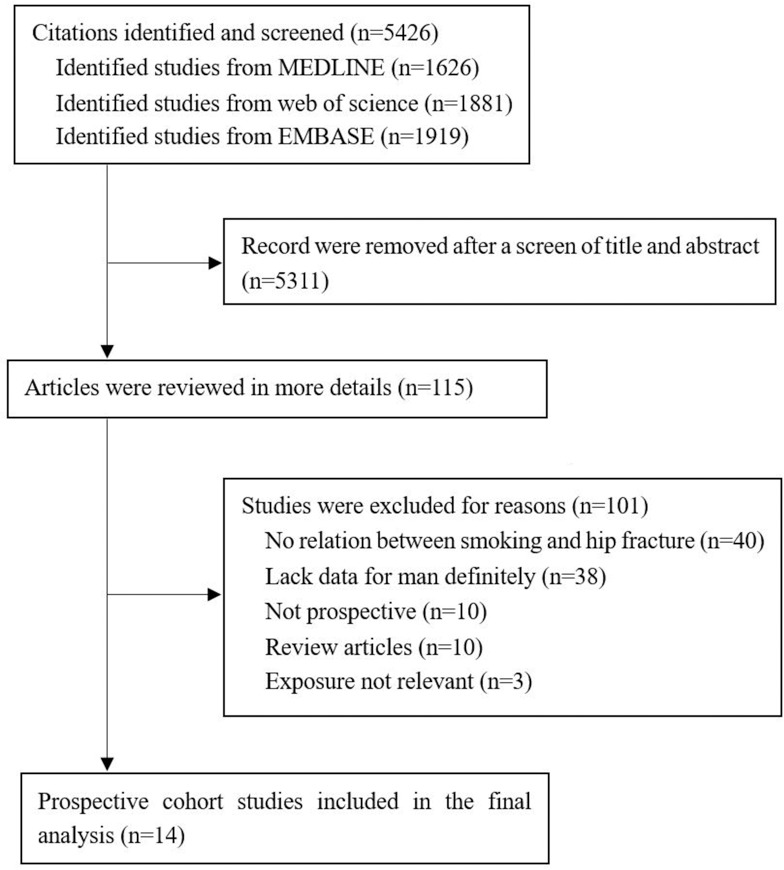
Study selection. Literature search for the meta-analysis.

The process used to select the studies and participants included in present meta-analysis is summarized in Fig 1. We initially searched 5426 potentially eligible studies, but most of them were excluded by title and abstract screen. After that, a total of 101 potential papers were excluded in more details. Finally, fourteen prospective cohort studies were included in present mate-analysis.

### Study characteristics

Characteristics of the 14 included cohort studies [3, 5–12, 16–20] were shown in Table 1. They were published from 1991 [5] to 2016 [18], and the sample sizes varied from 1412 [19] to 50000 [9]. The mean durations of follow-up ranged from 3 [8, 16] to 30 years [7]. Eight studies were conducted in Europe, five in U.S.A., and one in Singapore. Obviously, only one [6] RR from the study was not adjusted for anything while two others [5,11] just based on age, and the rest of these publications adjusted for multivariable potential factors relating to hip fracture, such as health, body mass index (BMI), smoking status, alcohol consumption, physical activity, chronic disease, calcium intake, calories, protein consumption, and so on. According to the nine-star Newcastle-Ottawa Scale, the quality scores of included studies ranged from 6 to 9. Most of them (12/14) is greater than or equal 7 stars. (Table 1)

**Table 1:** Summary of the characteristics of the included prospective cohort studies.

First author	Years	Location	Duration (years)	Size of cohort	Mean age (range)	Smoking status	No. of fracture patients	Adjusted relative risk (95% CI)	Study Quality	Adjustment for Covariates
Paganini-Hill	1991	U.S.A	7	5049	73	Former	50	1.16 (0.73–1.86)	7	Age
						Current	9	2.23 (1.04–4.8)		
						Current^a^	NA	1.94 (0.96–3.94)		
Meyer	1993	Norway	11	27015	35–49	Former	14	1.25 (0.56–2.81)	8	Age
						Current (1–14)	14	0.93 (0.41–2.09)		
						Current (≥15)	19	1.81 (0.84–3.89)		
Forsen	1994	Norway	3	18198	≥50	Current^a^	136	1.8 (1.2–2.9)	9	Age, leanness, ill health, physical inactivity, and self-reported.
Hemenway	1994	U.S.A	6	50000	40–75	Former	29	1.05 (0.61–1.81)	7	Alcohol consumption, BMI, height, and smoking status.
						Current	6	1.08 (0.44–2.67)		
Mussolino	1998	U.S.A	14	2879	≥45	Current	71	1.45 (0.86–2.42)	7	Alcohol consumption, chronic disease, calcium intake, calories, physical activity, protein consumption, self-reported, and smoking status.
Forsen	1998	Norway	3	14428	50–64	Former	4	2.3 (0.3–21)	7	Age, BMI, physical inactive, and subjective health.
						Current	11	4 (0.5–32)		
					65–74	Former	11	4.3 (1.0–20)		
						Current	13	5.3 (1.2–25)		
					≥75	Former	15	1.1 (0.5–2.3)		
						Current	18	1.6 (0.8–3.3)		
Hoidrup	2000	Denmark	5–13	17379	20–93	Current	316	1.59 (1.04–2.43)	8	Age, alcohol intake, BMI, menopausal age, physical activity, study of origin, and school education.
						Former	100	1.16 (0.74–1.83)		
Olofsson	2005	Sweden	30	2322	71	Current	96	3.03 (1.02–3.44)	8	Age, alcohol, BMI, cardiovascular disease, diabetes mellitus, leisure time physical activity, marital status socioeconomic class, and physical activity at work.
						Former	NA	1.87 (1.02–3.44)		
Holmberg	2006	Sweden	16	22444	44	Current^a^	163	2.20 (1.54–3.15)	7	Age, BMI, diabetes, smoking, and self-rated health.
Koh	2009	Singapore	7	27913	71.4	Former	80	1.27 (0.93–1.72)	6	Age, education, weekly vigorous work or strenuous sports, and year of recruitment.
						Current	107	1.23 (0.92–1.64)		
Stolee	2009	Canada	10	13773	81.5	Current	223	1.58 (1.03–2.42)	6	NA
Jutberger	2010	Sweden	3	1412	69–80	Current	38	2.34(0.97–5.65)	8	Age, BMD, BMI, calcium intake, center, glucocorticoid treatment, and physical activity.
									
									
Trimpou	2010	Sweden	30	7495	46–56	Former	86	1.06 (0.81–1.40)	8	Age, alcohol consumption, tall stature, low occupational class, interim stroke or dementia, and smoking.
						Current	234	1.58 (1.27–1.96)		
								
Jane	2016	U.S.A	8.6	5994	>65	Current	97	2.05 (1.05, 3.98)	7	Age, BMD, clinic, and race.

BMD, bone mineral density; BMI, body mass index; NA, not available.

^a^ Current smokers compared with nonsmokers which include never smokers and former smokers.

### Current smokers compared with never smokers

**Fig 2:**
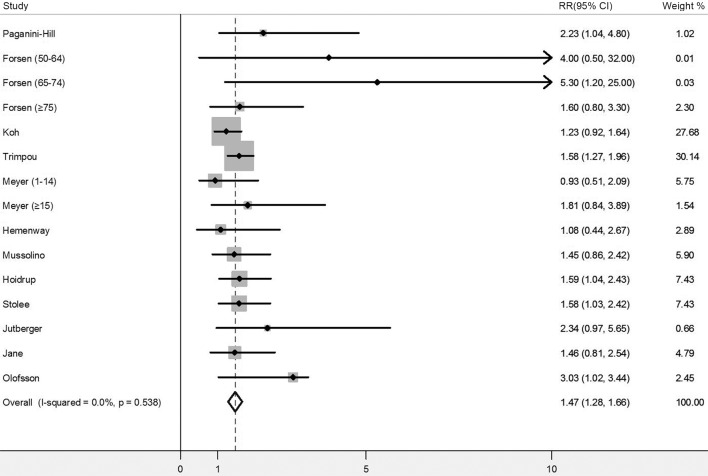
Meta-analysis of risk of hip fracture for current smokers compared with never smokers. RR, relative risk; CIs, confidence intervals.

Twelve of the included publications [3, 5–12, 18–20] reported the RRs for current smokers compared with never smokers. Five of them [3, 5–7, 20] on the association of cigarette smoking and risk of hip fracture showed a statistically significant positive association, and the remaining seven studies [8–12, 18, 19] yielded positive but not significant association. The pooled RRs for these twelve studies was 1.47 (95% CI, 1.28–1.66), and no evidence of heterogeneity was found across these publications (p = 0.538; *I*^2^ = 0%) (Fig 2). The result was consistent when perform the analyses omitting one study at a time as a sensitivity analysis. Publication bias was not found when detected by Begg’s test (p = 0.83) or Egger’s test (p = 0.92). In order to detect the potential factors that may have influenced the combined RRs for current smokers compared with never smokers, subgroup analyses were conducted according to study region (Europe, USA, and Asia), duration of follow-up (<10, ≥10 years), sample size (<20000, ≥20000 participants), and study quality (<8, ≥8 stars). All these subgroup analyses suggested no significant difference in results (Table 2).

**Table 2:** Subgroup meta-analyses for current smokers versus never smokers.

Subgroups	Number of studies	Relative risk (95% CI)	*I*^2^ (%)	p-Heterogeneity
Total	12	1.47(1.28, 1.66)	0	0.53
Geography region				
Europe	7	1.57 (1.27, 1.88)	8.6	0.36
North America	4	1.43 (0.94, 1.93)	0	0.78
Asia	1	1.23 (0.92, 1.64)	NA	NA
Length of follow-up				
< 10 years	6	1.28 (0.85, 1.60)	0	0.85
≥ 10 years	6	1.58 (1.14, 2.03)	28	0.2
Size of cohorts				
< 20000	9	1.64 (1.40, 1.88)	0	0.74
≥ 20000	3	1.27 (0.98, 1.56)	0	0.56
Study quality				
< 8	7	1.36(1.09, 1.62)	0	0.9
≥ 8	5	1.66 (1.20, 2.12)	41	0.1

NA, not available.

### Former smokers compared with never smokers

**Fig 3:**
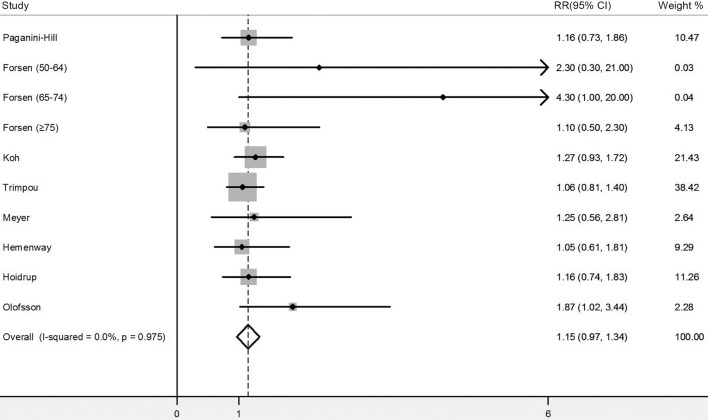
Meta-analysis of risk of hip fracture for former smokers compared with never smokers. RR, relative risk; CIs, confidence intervals.

Eight studies [3, 5, 7–11, 20] reported the RRs for former smokers compared with never smokers. Almost all these studies showed non-significant positive relationship between cigarette smoking and risk of hip fracture except for Olofsson.et.al [20]. The pooled adjusted RR was 1.15 (95% CI, 0.97–1.34), with no heterogeneity (*I*^2^ = 0%, p = 0.975) (Fig 3). No publication bias was found with Begg’s test (p = 0.78) or Egger’s test (p = 0.85).

### Current smokers compared with nonsmokers

**Fig 4:**
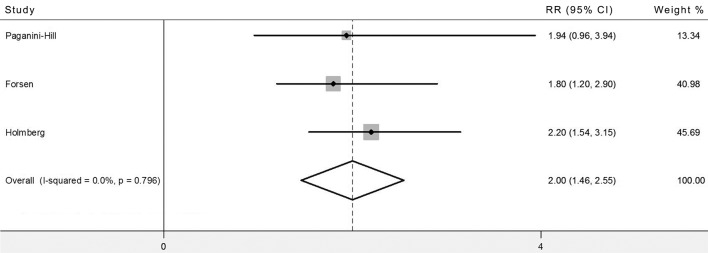
Meta-analysis of risk of hip fracture for current smokers compared with nonsmokers. RR, relative risk; CIs, confidence intervals.

Three studies [5, 16, 17] provided the RRs for current smokers versus nonsmokers, including former smokers and never smokers. The pooled RRs indicate that, compared with nonsmoker, current smokers suffer two-fold risk of hip fracture (RR = 2.00, 95% CI, 1.46–2.55) (Fig 4).

## Discussion

The present meta-analysis of 14 prospective cohort studies involving 216301 participants who do not receive any anti osteoporotic medication and 1922 hip fracture patients diagnosed by radiographic examinations in hospitals, confirming a positive association between cigarette smoking and risk of hip fracture in male. Current smokers had a 1.47-fold risk of suffering hip fracture compared with never smoker. However, the relationship between former smoker and risk of hip fracture does not exist a significant positive correlation. We also found that current smokers had a 2-fold risk of developing hip fracture compared with nonsmoker, however, only three studies were included in this subgroup meta-analysis, which could have biased the results. Our results are similar with a recent meta-analysis, which reported that, compared with never smokers, the pooled RR of hip fracture for current female smokers was 1.30 (95%CI, 1.16–1.45), and for former female smokers was 1.02 (95%CI, 0.93–1.11) [4].

Nowadays, the mechanism of the positive association between smoking and risk of hip fracture is unclear, however, several biological factors may underlie the association found in the present meta-analysis. Calcium is one of the key point maintaining bone health [21]. Smoking may reduce bone mass through reducing the level of 25-hydroxyvitamin D, which impaired the absorption of calcium and the metabolism of vitamin D. One possible reason for that was smoking may improve hepatic metabolism of vitamin D metabolites, following induction of liver enzymes [22]. Parathyroid hormone, changing the proliferation of bone cells as well, specifically, osteoblast and osteoclast, which may influence the absorption of calcium in our body and the metabolism of bone [23].

Low bone mineral density (BMD) has been recognized as one of the major causes of the increasing risk of osteoporosis and hip fracture, while the BMI of people are associated with the BMD [24]. Smoking often makes people thinner and with a lower BMI. One possible mechanism which cigarette smoking cause bone loss through its effect on changing body weight by suppressing the appetite of smokers [25], the article from Klesges et al study [26] found that the weight of smokers less than nonsmokers for approximately 7–8 pounds in middle age, which strengthen the evidence that cigarette smoking increases the risk of bone loss. Furthermore this is reported to be higher in male smokers than in female smokers in Hannan’s research [27], perhaps man experience a higher exposure to smoking than woman. In general, we observed a higher risk ratios of fracture for male than female, specifically for osteoporotic fracture [28].

Smoking has been proved to affect level of adrenal cortical hormones which are precursors of estrogen and testosterone [29]. Nicotine has been determined to have anti-estrogenic effects and decrease the production of estrogen [30, 31], therefore, comparing with nonsmoker, menopause would occur approximately 2 years earlier in female smokers [32] and the age of menopause was recognized as a significant indicator of osteoporosis [33]. However, although some articles support that the level of testosterone in male smokers is higher than nonsmokers, the testosterone tend to influence metabolism of bone in man has not been well defined than the relative effect of estrogen in woman [29,34].

Cigarette smoking is associated with increased level of free radicals, which may contribute to bone resorption. A prospective cohort study from Sweden [35] found that current smokers with a low intake of vitamin E or C may increase the risk of hip fracture, which the OR was 3.0 (95% CI1.6–5.4) and 3.0 (1.6–5.6) respectively. In contrast, the OR of hip fracture risk would drop to 1.1 (95% CI 0.5–2.4) with vitamin E and 1.4 (95% CI 0.7–3.0) with vitamin C when current smokers with a high intake of vitamin E or C, in addition, comparing with the nonsmokers, hip fracture risk was almost fivefold increased (OR 4.9 [2.2–11.0]) in smokers with low intakes of vitamins E and vitamins C, furthermore, a direct toxic effect on the bone cells and tissues by nicotine and non-nicotine components, which may reduce blood supply to the bone [29,36]. An increasing number of researches are needed to determine whether these mechanisms underlie smoking's effect on bone metabolism.

Similar to other meta-analysis, several limitations in present meta-analysis should be of concern. First, we were unable to examine the dose-response relationship between smoking and risk of hip fractures, as well as the risk of hip fracture since cessation of smoking, because smoking history and classification method of smoking cessation from the including studies were significantly different. Second, the adjustment for confounders of all the included articles are not the same, which may exaggerate or underestimate the results. However, 13 of the 14 included prospective cohorts adjusted for age, and over half adjusted for major potential confounders, including BMI, alcohol use, and so on. Third, the present meta-analysis is based on published researches, and publication bias may affect the results. However, no evidence of publication bias was found when evaluated by Begg’s test and Egger’s test. Finally, the study has a significant geographical differences. Our pooled result based on 13 western reports and one Singapore report, thus the generalization of the conclusion should be cautious.

The present meta-analysis also has some strengths. First, to our knowledge, this is the first meta-analysis about the association between cigarette smoking and risk of hip fracture in male. Second, all the included articles were prospective cohort studies in design, which strengthened the power and minimized recall and selection bias compared with case-control and retrospective cohort studies. Third, the sample sizes were large (1922 patients with hip fracture and 216301participants) and the sensitivity analysis was consist with our result, indicating our findings were reliable and robust. Finally, no evidence of heterogeneity was found across the included publications.

In short, the present meta-analysis of 14 prospective studies suggests that, compared with never smokers, cigarette smoking increase risk of hip fracture in man, specifically in current smokers. However, further larger prospective cohorts with more power or meta-analysis of individual patient data are needed to confirm this association.

## Supporting Information

S1 Table PRISMA-2009-Checklist-MS-Word. (DOC)
